# Mesenchymal Stem Cells for the Treatment of Idiopathic Pulmonary Fibrosis

**DOI:** 10.3389/fmed.2018.00142

**Published:** 2018-05-15

**Authors:** Argyrios Tzouvelekis, Rebecca Toonkel, Theodoros Karampitsakos, Kantha Medapalli, Ioanna Ninou, Vasilis Aidinis, Demosthenes Bouros, Marilyn K. Glassberg

**Affiliations:** ^1^First Academic Respiratory Department, Sotiria General Hospital for Thoracic Diseases, University of Athens, Athens, Greece; ^2^Division of Immunology, Alexander Fleming Biomedical Sciences Research Center, Athens, Greece; ^3^Department of Medicine, Florida International University Herbert Wertheim College of Medicine, Miami, FL, United States; ^4^Department of Surgery, University of Miami Miller School of Medicine, Miami, FL, United States; ^5^Department of Medicine, University of Miami Miller School of Medicine, Miami, FL, United States

**Keywords:** idiopathic pulmonary fibrosis, mesenchymal stem cells, treatment, safety, efficacy

## Abstract

Idiopathic pulmonary fibrosis (IPF) is an inexorably progressive lung disease of unknown origin. Prognosis is poor, with limited treatment options available, and the median survival remains just 3–5 years. Despite the use of pirfenidone and nintedanib for the treatment of IPF, curative therapies remain elusive and mortality remains high. Regenerative medicine and the use of cell-based therapies has recently emerged as a potential option for various diseases. Promising results of preclinical studies using mesenchymal stem cells (MSCs) suggest that they may represent a potential therapeutic option for the treatment of chronic lung diseases including IPF. Encouraging results of Phase 1 studies of MSCs various have reduced safety concerns. Nonetheless, there is still a pressing need for exploratory biomarkers and interval end-points in the context of MSCs investigation. This review intends to summarize the current state of knowledge for stem cells in the experimental and clinical setting of IPF, present important safety and efficacy issues, highlight future challenges and address the need for large, multicenter clinical trials coupled with realistic end-points, including biomarkers, to assess treatment efficacy.

## Introduction

Idiopathic Pulmonary Fibrosis (IPF) is a progressive debilitating lung disease of unknown etiology (1–4). The disease is characterized by a combination of histological changes including extracellular matrix (ECM) deposition, phenotypic changes of fibroblasts and alveolar epithelial cells, formation of fibroblastic foci, and scattered areas of aberrant wound healing interspersed with normal lung parenchyma (1, 5–14).

Current evidence suggests that the areas of fibrosis seen in lungs of patients with IPF share many features with normal aging lung, such as genomic instability, telomere attrition, mitochondrial dysfunction, cellular senescence, and immune dysregulation (10, 15, 16). Due to the inefficacy of immunomodulatory and immunosuppressive agents in the past, the role of the immune system in the pathogenesis of IPF remains poorly understood (17–22). However, highly activated and proliferative CD4+ cells and functional impairment of T regulatory cells (Tregs) in patients with IPF, suggest a link between immunity and pulmonary fibrosis (10, 23, 24).

There are two approved compounds for the treatment of IPF: pirfenidone and nintedanib. Pirfenidone is an antifibrotic compound with an unclear mechanism of action targeting several molecules including transforming growth factor-β (TGF-β), tumor necrosis factor-α (TNF-α), and interleukin 6 (25). Nintedanib is a tyrosine-kinase inhibitor, targeting vascular endothelial growth factor receptor (VEGFR), fibroblast growth factor receptor (FGFR), and platelet derived growth factor receptor (PDGFR) (22).While the use of pirfenidone and nintedanib have been shown to slow the progression of IPF (26–28), both compounds have significant side effects and neither is curative (28–30). Morbidity and mortality from IPF remains high and thus there is a pressing need for alternative therapeutic options for this complex disease (7, 31–33). The US National Institutes of Health database lists 493 complete or ongoing clinical trials of MSCs (34). Toward this end, regeneration and cell therapies such as the use of mesenchymal stem cells (MSCs) have emerged as a potential option.

MSCs are multipotent cells able to differentiate into a number of different cell lines and exert immunomodulatory, anti-proliferative, and anti-inflammatory effects. Their multipotency, migratory ability, and immunoprivileged state has led to extensive research efforts for therapeutic applications in several diseases including cardiac ischemia (35–39), ischemic acute renal failure (37), sepsis (40), autoimmune disorders (41), severe graft-vs. -host disease (42), pancreatic islet and renal glomerular repair in diabetes (43), fulminant hepatic failure (44), chronic lung diseases (45–48), and acute lung injury (49–53) (Table 2).

MSCs are easily harvested from many tissues (peripheral blood, adipose tissue, bone marrow, and umbilical cord) and may be expanded *in vitro* with minimal modifications. MSCs represent the most extensively studied stem cell population (54). Research supports the immunomodulatory, anti-inflammatory, and potentially anti-fibrotic properties of MSCs (49, 55, 56). Importantly, MSCs are “immune privileged,” lacking expression of class II major histocompatibility complex (MHC-II). Therefore, allogeneic use of MSCs is possible (57).

## Preclinical studies

Recent studies on the pathophysiology of IPF suggest that early alveolar injury activates abnormal alveolar epithelial cells and stimulates the release of mediators including matrix metalloproteinases and TGF beta-1 (2, 58–61). These mediators activate cytokines and chemokines including IL-1 and IL-13 leading to the phenotype of abnormal wound healing (62–65). Therefore, IPF is considered a complex and multifactorial disease characterized by alveolar epithelial injury and alveolar collapse, fewer alveolar epithelial type II cells, alveolar stem cell exhaustion, and myofibroblast deregulation due to living on a fibrotic matrix (66, 67).

Several experimental studies have been conducted in order to investigate the effect of MSCs from various organs, mainly from bone marrow with a dosage ranging between 0.1 × 10^6^ and 4 × 10^6^ cells, in pathways associated with lung injury and pulmonary fibrosis and several end-points had been set (68, 69) (Table 1). The majority of studies recorded substantial improvement in histopathology (56, 64, 70–82), decrease to Ashcroft score (70–72, 77, 79, 80, 83) and lung collagen content (56, 70–81), reduced pulmonary transforming growth factor-b (TGFb) levels (56, 70–81, 84) and decreased BAL neutrophil count (76, 77, 80, 81, 85) following to MSCs administration. Importantly, both bone marrow and amnion-derived MSCs reduced TGFb levels (84). However, to this end, data are still conflicting regarding levels of tumor necrosis factor-a (TNF-a) (56, 71, 83–85), interleukins IL-1, IL-6 (71, 77, 81, 84, 86) and metalloproteinases MMP-2, MMP-9, MMP-13 (56, 71, 83, 84) following administration of MSCs (4, 87–89).

**Table 1:** Main results of preclinical studies of mesenchymal stem cell therapy in experimental pulmonary fibrosis based on end-points set.

**End-point**	**Outcome**	**Studies**
Histopathology	Significant improvement	(56, 64, 70–82)
Ashcroft score	Decrease	(70–72, 77, 79, 80, 83)
Lung collagen content	Decrease	(56, 70–81)
TGF-b	Decrease	(56, 70–81, 84)
BAL neutrophil count	Decrease	(76, 77, 80, 81, 85)
TNF-a	Conflicting	(56, 71, 83–85)
IL-1, IL-6	Conflicting	(71, 77, 81, 84, 86)
MMP-2, MMP-9, MMP-13	Conflicting	(56, 71, 83, 84)
Survival compared with pirfenidone	Improved	(64)

*BAL, bronchoalveolar lavage, IL, interleukin, MMP, metalloproteinases, TGF-b, transforming growth factor-beta, TNF-a, tumor necrosis factor-a*.

**Table 2:** Results of clinical human studies of mesenchymal stem cell therapy.

**Study**	**Disease model**	**Cell type**	**Delivery and dose**	**Safety results**	**Efficacy results**
(42)	Acute graft versus host disease	Allogeneic BM-MSCs; HLA matched and mismatched	IV, 1.4 × 10^6^ cells/kg	No adverse effects reported	Complete response in 30 of 55 patients. Partial response in 9 of 55 patients
(37)	Myocardial infarction	Allogeneic BM-MSCs; Non-HLA matched	IV, 0.5, 1.6, or 5.0 × 10^6^ cells/kg	No difference in adverse events compared with placebo	Decreased arrhythmic events. Decreased PVCs. Improved post-event ejection fraction. Improved overall clinical status. Improved FEV_1_ percent predicted
(41)	Refractory lupus	Allogeneic BM-MSCs; Non-HLA matched family members	IV, 1 × 10^6^ cells/kg	No adverse events reported	Improved SLE disease activity index score. Improved 24-h proteinuria
(112)	Ischemic cardiomyopathy	Allogeneic vs. autologous BM-MSCs; Non-HLA matched (allogeneic)	Endocardial, 20, 100, or 200 × 10^6^ cells	One patient in each arm hospitalized for heart failure. No statistically significant difference in adverse events between arms	Improvement in 6MWT and QOL index with autologous MSCs. CT evidence of reverse LV remodeling in both arms. Improved LV and diastolic volumes with allogeneic MSCs
(110)	COPD	Allogeneic BM-MSCs; Non-HLA matched	IV, 100 × 10^6^ cells/ infusion Four monthly infusions	No difference in adverse events compared with placebo	No effect seen on frequency of COPD exacerbation or PFTs. Decreased circulating C-reactive protein in patients with high baseline levels
(103)	IPF	ADSCs-SVF	Endobronchial, 0.5 × 10^6^ cells/kg of body weight in 10cc; 3 dosages over 3 months	No difference in adverse events compared with placebo. No ectopic tissue formation	Cell-treated patients did not deteriorate in both functional parameters and indicators of quality of life
(109)	IPF	Allogeneic placental MSCs	IV, 1 & 2 × 10^6^ cells/kg; one dose	Minor and transient acute adverse events	Stable lung function. No evidence of worsening fibrosis
(53)	ARDS	Allogeneic BM-MSC	IV, 1, 5, or 10 × 10^6^ cells/kg; 3 patients per dosage arm	No adverse events Serious adverse events after infusion (3 patients), non-MSC related	None
(105)	IPF	Allogeneic BM-MSC	IV, one dose: 20 × 106 (*n* = 3) 100 × 106 (*n* = 3) and 200 × 106 cells (*n* = 3)	No treatment-emergent serious adverse events. Two non-treatment related deaths due to progression of IPF	(Exploratory results): 3.0% mean decline in % predicted FVC and 5.4% mean decline in % predicted DLCO
(113)	IPF	ADSCs-SVF	Endobronchial, 0.5 × 10^6^ cells/kg of body weight in 10cc; 3 dosages over 3 months	No difference in adverse events compared with placebo. No ectopic tissue formation	Median overall progression-free survival 26 months. Median overall survival 32 months. All patients alive for at least 2 years after first administration

*ADSCs-SVF, autologous adipose derived stromal cells-stromal vascular fraction; BM-hMSCs, human bone marrow-derived mesenchymal stem cells; IPF, Idiopathic pulmonary fibrosis; PD-MSCs, placenta-derived mesenchymal stem cells*.

The majority of studies investigating the effect of donor MSCs on BLM-induced pulmonary fibrosis have used young male mouse models (90, 91). Young mice, however, undergo spontaneous resolution of BLM-induced pulmonary fibrosis in some studies (69, 90, 91). Although IPF is primarily a disease of individuals over the age of 50, most studies have also utilized young female mice to evaluate the molecular patterns and potential therapeutic targets for patients with IPF (92–94). Interestingly, in one study of bleomycin induced fibrosis in mice, MSCs were found to improve survival when compared with pirfenidone (64). This study also reported downregulation of IL-2, IL-1b, TNF-α, and TGFβ leading to a reduction in inflammation (64). In addition, downregulation of MMPs was noted with a reduction in collagen deposition and fibrosis (64).

Collectively, MSCs seem to exert pleiotropic effects in the site of lung injury including anti-inflammatory, immunomodulatory, antifibrotic effects (65), engagement in paracrine signaling (95), activation of resident stem cells, and differentiation into local cell types (56, 65, 74, 77, 79, 83, 85, 96–98). Preclinical studies have shown MSCs to be efficacious in the treatment and prevention of lung fibrosis (65, 69). Nonetheless, concerns remain regarding the activity of MSCs within a pro-fibrotic microenvironment (99–102). While some preclinical studies suggest that MSCs might promote fibrosis, to date, no human studies have found a similar pro-fibrotic effect (37, 42, 91, 100, 101, 103–112).

## Clinical trials

Early clinical studies of MSCs in patients with IPF have shown promising safety profiles (30, 103, 114). Phase 1 clinical trials have been conducted for safety of MSC therapy. A phase Ib study of endobronchially administered autologous adipose-derived MSCs showed not only acceptable safety outcomes, but also improvements in quality of life parameters (103). The recently published longitudinal outcomes of this study also demonstrated an acceptable safety profile, 100% survival rate 2 years after first administration and a median overall progression-free survival of 26 months (113). Furthermore, studies of intravenously administered placental derived MSCs (105, 109) found that administration of up to 2 × 10^6^ cells per kilogram was safe in subjects with moderately severe IPF (109). Importantly, the authors reported only minor and transient alterations in peri-infusion hemodynamics and gas exchange, reducing the concerns for embolization of stem cells to an already compromised pulmonary vasculature. Subjects were followed for six months with no observed decline in forced vital capacity (FVC), diffusing lung capacity for carbon monoxide (DLCO), six-minute walk test (6MWT), or CT fibrosis score (90). The AETHER trial also showed favorable safety outcomes for the intravenous delivery of a single dose of allogeneic MSCs in patients with IPF up to 2 × 10^8^ cells (105). Although this was an underpowered study for the detection of significant changes in functional indices, the mean decline in % predicted FVC and DLCO were below the thresholds for disease progression (1, 115). ReCell, an FDA approved phase 1b multidose, randomized, double-blind trial of 10 × 10^6^ cells delivered intravenously to patients with IPF, has not yet begun enrollment.

## Outstanding challenges

While it now appears that it is safe to use MSCs in patients with IPF, many questions and challenges remain. In the preclinical realm, there is a need for animal models more representative of chronic IPF (91, 116) for the continued study of how MSCs exert their effects. Bleomycin induced pulmonary fibrosis is still considered the best available animal model for preclinical testing (91, 117). However, there is increasing criticism that potential therapies usually administered the first 7 days following bleomycin exposure may act mainly through prevention of the inflammatory cascade rather than reversal of fibrosis, thus limiting their applicability to human IPF (69). First, improved animal models will enable the identification of biomarkers that may be useful as measures of disease activity and/or treatment effect. Second, the timing of treatment for best effect needs to be better elucidated. Furthermore, the most efficacious source of MSCs and the role of age need to be more fully explored. One report suggesting that adipose-derived MSCs from young, but not old, mice prevent bleomycin induced lung fibrosis in an aged mouse model (118) highlights the need for further research in this area.

Several challenges in the clinical setting also remain to be addressed. The optimal source of MSCs, the best route of administration, the number and timing of administrations, and the appropriate dosing interval. Thus, allogeneic human bone marrow-derived and autologous adipose derived MSCs have been the most studied in the context of IPF. There are limited studies on endogenous stem cells from the lungs of patients with IPF and concerns remain surrounding the risk of biopsy and the potential for intervention-induced IPF exacerbation and the possibility of detrimental effects on lung function from biopsy. Lung tissue obtained at the time of lung transplant remains the best tool for study.

It is also critical to characterize appropriate endpoints to assess treatment effectiveness in these patients (100, 119–129). Molecular biomarkers would be the optimal choice for the assessment of cell based therapies. Finally, well-designed and meticulously conducted multicenter randomized clinical trials of MSCs for the treatment of IPF are needed to assess efficacy.

## Moving forward

While preclinical trials suggest that MSCs may be effective in the treatment of IPF, and early clinical trials suggest that they are likely to be safe in the population, insufficient data exists at this time to definitely state that the use of MSCs for the treatment of IPF is either safe or efficacious. Despite this lack of evidence, cell based therapies are being aggressively marketed to this vulnerable patient population. A recent study found that as of August of 2016, there were at least 351 stem cell related businesses registered in the United States. These sites offer unproven, experimental treatments for a wide variety of conditions (130, 131). In the case of IPF, desperate patients and their physicians continue to succumb to an onslaught of marketing and branding of as yet unproven “stem cell” treatments. Unfortunately, these businesses are also almost wholly unregulated (132). Publication of case reports of harm arising from the misuse of unproven treatments support increased government oversight in the interest of patient safety.

## Conclusion

Idiopathic Pulmonary Fibrosis (IPF) is a debilitating lung disease characterized by a progressive decline in lung function ultimately resulting in death. The lack of curative treatments for this disease has created an urgency for other potential therapeutic options. Preclinical studies suggest that because MSCs have immunomodulatory, anti-inflammatory, and potentially anti-fibrotic properties, they may be efficacious in the treatment of IPF. Early clinical trials have shown that MSCs may be safely administered to patients with IPF, but large multicenter randomized trials still need to be performed.

## Author contributions

AT, RT and TK wrote the initial manuscript. The manuscript was supervised and significantly modified by MG, AT, DB. KM, IN, VA offered significant intellectual contribution. All authors approved the final form of the manuscript.

### Conflict of interest statement

The authors declare that the research was conducted in the absence of any commercial or financial relationships that could be construed as a potential conflict of interest.
